# Bond strength to eroded dentin as per chlorhexidine use for controlling erosive wear or interface aging: an 18-month assay

**DOI:** 10.1590/1807-3107bor-2025.vol39.003

**Published:** 2025-01-13

**Authors:** Aloísio de Melo FARIAS-NETO, Karin LANDMAYER, Giovanni Aguirra LIBERATTI, Carlos Alberto Kenji SHIMOKAWA, Linda WANG, Heitor Marques HONÓRIO, Adriana Bona MATOS, Luciana Fávaro FRANCISCONI-DOS-RIOS

**Affiliations:** (a) Universidade de São Paulo – USP, School of Dentistry, Department of Operative Dentistry, São Paulo, SP, Brazil.; (b) Universidade de São Paulo – USP, Bauru School of Dentistry, Department of Operative Dentistry, Endodontics and Dental Materials, University of São Paulo, Bauru, SP, Brazil.; (c) Universidade de São Paulo – USP, Bauru School of Dentistry, Department of Pediatric Dentistry, Orthodontics and Public Health, Bauru, SP, Brazil.

**Keywords:** Tooth Erosion, Prevention, Chlorhexidine

## Abstract

The aim of this study was to assess the effect of a chlorhexidine digluconate solution (CHX) applied as an antiproteolytic agent for controlling erosive tooth wear or as part of the adhesive treatment on long-term bond strength to eroded dentin. Dentin specimens were abraded with a 600-grit silicon carbide (SiC) paper for 1 min (sound dentin - S), subsequently treated with 2% CHX for 1 min (with excess removed, followed by a 6-hour rest), and eroded by exposure to Coca-Cola for 5 min, three times a day, for 5 days (CHX-treated and eroded dentin - CHXE), or only eroded (eroded dentin - E). The specimens were acid-etched (15 s), rinsed (30 s), dried (15 s), and rehydrated with 1.5 μL of distilled water for 1 min, with excess removed (control - S.C/CHXE.C/E.C) or 2% CHX (S.CHX/CHXE.CHX/E.CHX). Adper Single Bond 2 was scrubbed twice on the surface for 15 s each and then light-cured for 10 s, and resin composite cores were built up. Specimens were sectioned into beams and microtensile bond strength was tested (μTBS; 0.5 mm/min) immediately or after 18-month aging. Failure modes were analyzed using a digital microscope. Data (μTBS/MPa) were analyzed by three-way ANOVA, followed by Tukey’s test (α = 0.05). μTBS to E and CHXE, irrespective of the rehydration solution and aging period, were equivalent to each other and lower than that to S. CHX as the rehydration solution reduced immediate and long-term µTBS to S. Aging reduced μTBS. By controlling tooth wear or interface aging, CHX could not influence long-term bonding to eroded dentin.

## Introduction

Eroded dentin has been associated with low bond strength to resin-based materials,^1,2^ especially after aging.^3^ Acidic challenges act on dentin initially releasing minerals from the interface between the peritubular and intertubular dentin. The challenges then demineralize the peritubular dentin and enlarge the dentinal tubules. Finally, acids act upon the intertubular dentin, making it rough and porous, resulting in a demineralized organic matrix over a partially demineralized area in the remaining mineralized dentin.^4^ Therefore, the hybrid layer established therein is thicker than that in sound dentin, which is attributed to inappropriate impregnation of resin monomers.^2^ The hybrid layer is structurally imperfect and porous and contains areas not fully reinforced by resin. Moreover, excessive moisture may prevent the complete conversion of monomers into polymers.^2^ These factors minimize the immediate effectiveness of bonding and make the interface more susceptible to degradation.^2,5^ Host proteases, especially metalloproteinases (MMPs) and cysteine cathepsins (CCs), then participate in the degradation of the extracellular matrix.^8^

For now, the most effective method for improving microtensile bond strength (μTBS) to eroded dentin, making it comparable to that of sound dentin, involves roughening the surface with a diamond bur, which seems to remove the demineralized dentin.^2^ The ultramorphological characteristics of the adhesive interface in eroded dentin roughened with a diamond bur hardly differ from those established in the same substrate treated with other methods (pumice cleaning, air abrasion, silicon polishing, or proxo-shape filing). The advantages of using a diamond bur become more evident over time.^2^ However, noninvasive or microinvasive alternatives have been studied to increase bond strength, considering that they should always be the first choice in clinical practice.^2,9^

Given that chlorhexidine digluconate (CHX) is a potent and nonspecific inhibitor of proteolytic enzymes,^10^ helping to maintain the stability of adhesive interfaces in dentin^11^ and controlling the progression of dentin erosive wear,^21-23^a study evaluated the effect of applying CHX solutions at different concentrations as an antiproteolytic primer during adhesive treatment, as well as μTBS to the eroded dentin in the immediate and medium term.^24^ Subsequently, they evaluated the duration of the effect of 2% CHX solution.^25^ Although CHX minimizes the degradation of the adhesive interface only at this concentration and only in the medium term, μTBS to the eroded dentin did not match those observed for sound dentin, either immediately or after aging for six or 12 months.

As an antiproteolytic agent, inhibiting MMPs and CCs,^26^ at a 0.012% concentration in gel form, CHX fully inhibited erosive wear, outperforming fluoride.^27^ Scanning electron microscopy evaluations confirmed the absence of wear on CHX-treated dentin and showed particle deposition within the dentinal tubules, likely due to the binding of CHX to apatite calcium.^10,28^ As with green tea extract used in similar contexts,^29,30^ preventing enzymatic degradation of the organic matrix controls demineralization by hindering ionic diffusion.^31^

We thus hypothesized that dentin treated with CHX prior to an erosive challenge, to prevent the progression of erosive wear, would positively influence the establishment of adhesive interfaces on eroded dentin, given the need to restore the lost dental structure for functional or aesthetic reasons.^32,33^ In addition, CHX could perhaps contribute to the longevity of the interface against aging even if it is not applied as part of the adhesive treatment. Studies are needed to evaluate strategies for increasing the durability of bonding to eroded dentin, particularly those involving protease inhibitors.^1,9,34^

This study aimed to explore the effect of a CHX applied as an antiproteolytic agent for controlling erosive tooth wear or as part of the adhesive treatment on immediate and long-term μTBS to eroded dentin when compared with sound dentin. The null hypotheses were that (a) the dentin substrate (sound - S, CHX-treated and eroded - CHXE, or eroded - E); (b) the antiproteolytic primer applied for dentin rehydration after acid-etching, washing, and air-drying (distilled water/control - C, or CHX); and (c) the aging of specimens (immediate testing - I or 18-month aging - 18 m) would not influence the outcomes.

## Methods

### Experimental design

This in vitro study was designed (Figure) to evaluate µTBS of a simplified etch-and-rinse adhesive system plus a nanoparticle resin composite to the dentin substrate at three levels (sound dentin - S, CHX-treated and eroded dentin - CHXE, or eroded dentin - E), rehydrated after phosphoric acid etching, rinsing, and air drying with an antiproteolytic primer at two levels (distilled water/control - C, or CHX), over time at two levels (immediately - I or after 18-month aging - 18m). The fracture pattern of the tested beams was analyzed under a digital microscope (50× magnification).

**Figure:**
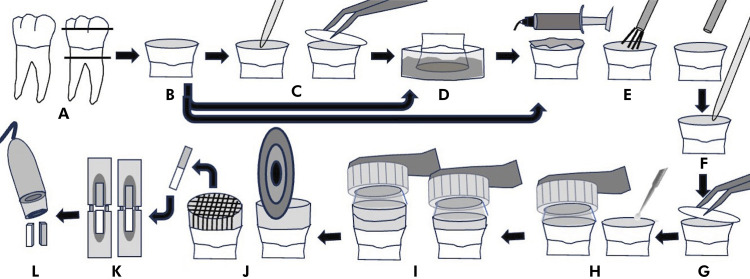
Experimental design: A) Third molars were obtained and exposed dentin was B) abraded with 600-grit SiC paper for 1 min (sound dentin - S), C) subsequently treated with 2% CHX for 1 min (excess removal, and 6-hour rest) and D) eroded by Coca-Cola for 5 min, three times a day, for 5 days (CHX-treated and eroded dentin - CHXE), or only eroded (eroded dentin - E). The specimens were then E) acid-etched (15 s), rinsed (30 s), dried (15 s), and F) rehydrated with 1.5 μL of distilled water (control - S.C/CHXE.C/E.C) or 2% CHX (S.CHX/CHXE.CHX/E.CHX), followed by G) excess removal. H) Adper Single Bond 2 was scrubbed twice on the surface for 15 s each, and light-cured for 10 s, and I) resin composite cores were built up. J) Specimens were sectioned into beams, and K) microtensile bond strength was tested (μTBS; 0.5 mm/min) immediately or after 18-month aging. L) Failure modes were analyzed using a digital microscope.

### Specimen preparation

Healthy extracted human third molars were provided by Biobank - Human Teeth Division at the main authors’ institution - after approval of the research protocol by the Research Ethics Committee (CAAE 90202918.6.0000.0075; Assents #2.097.260 and #3.612.543).

A sample size of eight specimens per group was calculated, considering an α error of 5%, a β error of 20%, a standard deviation of 5.4,^24^ and an effect size of 12 MPa.

Coronal occlusal thirds of the third molars were sectioned (Isomet Low Speed Saw; Buehler, Lake Bluff, USA), and the exposed midcoronal dentin was abraded with a water-cooled 600-grit SiC paper for 1 min for smear layer standardization. The smear-layered dentin was categorized as sound dentin (S, n = 16). CHX-treated and eroded dentin (CHXE, n = 16) was prepared by sequentially applying a 2% CHX solution (1.5 µL), and excess solution was removed with absorbent paper after 1 min, followed by a 6-hour rest period).^21^ Thereafter, the specimens were subjected to erosive pH cycling. Eroded dentin (E, n = 16) was prepared only by erosive pH cycling. pH cycling was performed by exposing the dentin to a cola-based soft drink (Coca-Cola, Spaipa, Marília, Brazil; pH 2.6) at room temperature. Each specimen was immersed in 60 mL of the drink for five min, three times a day, for five days.^24,25^ Specimens were stored in daily-renewed artificial saliva (pH 7.0; 60 mL per specimen) for 12 h prior to cycling and between erosive challenges.

After 15 s of acid-etching with 37% phosphoric acid gel (Condicionador Dental Gel, Dentsply, Rio de Janeiro, Brazil), 30 s of water rinsing, and 15 s of oil- or water-free air-drying (approximately 3 cm from the surface to keep the dentin dry), half of the specimens in each group had their surfaces rehydrated with 1.5 µL of distilled water (S.C, CHXEC, and EC, n=8). The remaining half of the specimens were rehydrated with 1.5 µL of a 2% CHX solution (S.CHX, CHXE.CHX, and E.CHX, n = 8). After 1 min, excess solution was removed with absorbent paper, keeping the dentin moist. Adper Single Bond 2 (3M ESPE, St. Paul, USA) was continuously scrubbed on the dentin surface for 15 s, applied twice, and solvent evaporation was aided by a gentle air stream. Light-curing was performed for 10 s (Radii-cal, SDI Limited, Bayswater, Australia). The mean radiant exitance was 794.7 mW/cm^2^, measured from five readings using an integrating sphere coupled to a spectrometer (MSC 15-W; Gigahertz-Optik, Türkenfeld, Germany) and considering an area of 6.9 mm of light emission. Finally, two increments (each around 2 mm in thickness) of a resin composite (Filtek Z350, shade A2; 3M ESPE, St. Paul, USA) were individually built up and light-cured for 20 s using the same device, as previously described.

### Microtensile bond strength test

After 24 h of storage in distilled water (37°C), each specimen was sectioned into a series of approximately 0.9 mm x 0.9 mm beams. Half of the beams were tested immediately, whereas the remaining beams underwent 18 months of aging in artificial saliva (37 °C; replacement every 14 days). Each beam was attached (Cola Almasuper Kit MDF, Almata Química, Curitiba, Brazil) to a custom-made Geraldeli’s testing jig and subjected to a tensile load (50 Kgf load cell) perpendicular to the adhesive interface at a crosshead speed of 0.5 mm/min until failure (Instron 5942; Instron Industrial Products, Grove City, USA).

Both fractured surfaces of each beam were analyzed under a digital microscope (Dino-Lite Digital Microscope; AnMo Electronics, New Taipei City, Taiwan) at 50× magnification. Failure modes were then classified as adhesive (A, failure only at the adhesive interface), mixed (M, presence of dentin or resin adjacent to the adhesive interface), cohesive in dentin (CD, failure in dentin), and cohesive in composite (CC, failure in composite).

### Statistical analysis

μTBS values were organized considering each tooth (n = 8) as an experimental unit. Three-way ANOVA and Tukey’s test were applied (Statistica 13.5.0.17; TIBCO Software, Palo Alto, USA), and the significance level was set at p ≤ 0.05.

## Results

All of the analyzed variables had a significant impact on the outcomes (p < 0.001 for substrate condition, rehydration solution, and aging). The interaction between substrate condition and rehydration solution was statistically significant (p < 0.001), while the other two-way interactions and the three-way interaction were not statistically significant (substrate*time p = 0.178; rehydration solution*time p = 0.738; substrate*rehydration solution*time p = 0.163). The post-hoc test, focusing on the only statistically significant interaction (substrate*rehydration solution), revealed differences between E.C and S.C (p < 0.001), E.C and S.CHX (p = 0.001), E.CHX and S.C (p < 0.001), E.CHX and S.CHX (p = 0.001), S.C and S.CHX (p < 0.001), S.C and CHXE.C (p < 0.001), S.C and CHXE.CHX (p < 0.001), S.CHX and CHXE.C (p = 0.010); but not between E.C and E.CHX (p = 1.000), E.C and CHXE.C (p = 0,971), E.C and CHXE.CHX (p = 1.000), E.CHX and CHXE.C (p = 0.981), and CHXE.C and CHXE.CHX (p = 0.964).

µTBS to E and CHXE, irrespective of the rehydration solution and aging, were comparable to each other and lower than to S. CHX as the rehydration solution reduced µTBS to S, both immediately and after aging. Aging unrestrictedly reduced µTBS (Table 1).

**Table 1:** Mean±standard deviation µTBS (MPa) to sound dentin (S), CHX-treated and eroded dentin (CHXE), and eroded dentin (E), according to the application of the post-etching rehydration solution (distilled water - C or CHX) and to aging (immediate testing - I or storage in artificial saliva for 18 months – 18 m).*

Variable	C		CX	
	I	18 m	I	18 m
S	49.5 ± 7.7^Aa†^	21.0 ± 6.3^Aa‡^	33.7 ± 8.9^Bb†^	12.8 ± 2.6^Bb‡^
CHXE	24.8 ± 7.9^Cc†^	6.8 ± 2.1^Cc‡^	24.7 ± 8.0^Cc†^	3.4 ± 0.5^Cc‡^
E	25.9 ± 6.1^Cc†^	2.3 ± 0.6^Cc‡^	26.9 ± 9.2^Cc†^	1.6 ± 0.5^Cc‡^

*Different superscript uppercase letters indicate statistically significant differences (p < 0.05) in each column for the variable “CHX application for controlling erosive tooth wear” (S, CHXE or E). Different superscript lowercase letters indicate statistically significant differences (p < 0.05) in each row for the variable “CHX application as part of the adhesive treatment” (C or CHX), at each aging time (immediate testing or storage in artificial saliva for 18 months). Different superscript symbols indicate statistically significant differences (p < 0.05) in each row for the variable “aging” (I or 18 m), in each post-etching rehydration solution (C or CHX). n = 8 in each table cell.

Adhesive and mixed failures were more prevalent than cohesive failures, either in dentin or composite under all experimental conditions (Table 2).

**Table 2:** Percentage (%) of adhesive (A), mixed (M), cohesive in composite (CC), and cohesive in dentin (CD) failures for sound dentin (S), CHX-treated and eroded dentin (CHXE), and eroded dentin (E) according to the application of the post-etching rehydration solution (distilled water - C or CHX) and to aging (immediate testing - I or storage in artificial saliva for 18 months – 18 m).

Variable	C		CHX	
	I	18 m	I	18 m
S				
A	18.8	50.0	17.1	45.0
M	58.3	23.9	68.3	25.0
CC	8.3	8.7	7.3	17.5
CD	14.7	17.4	7.3	12.5
CHXE				
A	34.1	54.3	56.8	65.6
M	43.2	22.9	31.8	21.9
CC	9.1	8.6	0.0	9.4
CD	13.6	14.3	11.4	3.1
E				
A	29.4	72.2	33.3	67.7
M	58.8	11.1	48.5	16.1
CC	8.8	5.6	9.1	6.5
CD	2.9	11.1	9.1	9.7

## Discussion

The null hypotheses were rejected, considering that the dentin substrate, the antiproteolytic primer applied for dentin rehydration after acid-etching, rinsing, air-drying, and aging of specimens significantly affected the outcomes.

While not all the characteristics of eroded dentin can be reproduced in vitro, the current literature underscores the need for a better understanding of the mechanisms involved in its effective and durable restoration with adhesive materials.^9^ Restoration may be the best choice to manage exposed and sometimes hypersensitive dentin and to restore function and aesthetics of compromised teeth.^32^ Moreover, restoration can prevent further progression of erosive tooth wear.^2^

Noninvasive strategies are, however, preferred, not only to prevent additional tissue loss but also to avoid concerns about low-quality adhesive interfaces on eroded dentin.^2^ In this respect, Charone et al. (2014)^35^highlighted the frequent use of mouthwashes containing biguanide (e.g., cationic CHX) provides benefits other than helping to treat gingivitis and periodontitis and reducing dental caries. Such benefits include preventing collagen fibril degradation by collagenases, which can either increase the longevity of adhesive restorations^11-20^or reduce dentin erosive wear.^21-23,30^ The association of both possibilities suggests the application of CHX to control dentin erosive wear would also influence the quality and longevity of the hybrid layer.

Regardless of aging, bond strength to CHX-treated and eroded dentin was comparable to that of eroded dentin, irrespective of whether CHX was applied as part of the adhesive treatment. This suggests that 2% CHX solution may not effectively minimize or prevent the degradation of collagen fibrils and, consequently, the progression of erosive wear by inhibiting local proteinases.^3,12,13,21,36^ These findings may be explained by the similarity between the substrates.

On the other hand, CHX might have minimized or prevented the degradation of collagen fibrils and, consequently, erosive wear.^13,21,29,30,36^ The concentration used was higher than the minimum inhibitory concentration for MMPs -2, -8, and -9 (≤ 0.0001%, 0.01-0.02%, and 0.002%),^10^ and the inhibition of CCs is dose-dependent.^26^ The efficacy of CHX as an antiproteolytic agent potentially depends not only on its deposition within the tissue but also on its substantivity, i.e., its capacity to remain retained on the substrate.^29,30^

It is possible to infer that resin monomers did not effectively impregnate the thick layer of collapsed fibrils. Additionally, the moisture of this layer impaired the conversion of monomers into polymers,^5,7,15^ limiting the bonding quality, even for CHX-treated and eroded dentin. Regardless of the thickness of the exposed fibril layer, the impaired bonding to dentin subjected to pH cycling, whether previously treated with CHX, can be explained by mineral loss at the interface between intertubular and peritubular dentin and the subsequent widening of tubule openings.^4^ This results in a reduced area for establishing the hybrid layer^2^ and increased moisture within the adhesive interface.

Studies assessing CHX as an antiproteolytic agent for controlling dentin erosive wear measure surface loss either quantitatively by profilometry or qualitatively by scanning electron microscopy.^17,37,38^ It is thus not possible to identify or quantify the demineralization of the substrate. Moreover, it is suggested that CHX-treated and eroded dentin interacts with restorative adhesive materials in a manner similar to caries-affected dentin.^1,9,20^ Even without tissue loss, dentin demineralization caused by erosive challenge seems sufficient to impair with the bonding of a simplified etch-and-rinse adhesive combined with a nanoparticle resin composite.

Nevertheless, strategies to limit or slow the progression of erosive wear can be advantageous even if diamond bur preparation of the dentin is still required for adequate bonding to the restorative material by effectively removing the demineralized layer.^2^ This approach allows removing a more superficial portion of the substrate, thereby preserving as much dental structure as possible.

CHX application as part of the adhesive treatment for eroded dentin has shown no beneficial effects, neither in this study nor in previous research.^24,25^ In addition, CHX has been shown to negatively affect the bond strength to sound dentin in the immediate term and in the long term. It is possible that CHX and the methacrylate functional copolymer of polyacrylic and polyitaconic acids present in Adper Single Bond 2 may have competed for bonding with calcium in the hydroxyapatite of the substrate. This hypothesis further elaborates on the role of 10-MDP in Adper Single Bond Universal.^18^ Similarly, hydrogen ions promote the interaction between polyacrylic acid and dentin collagen, which is also a target for CHX binding.^19^ These interactions would be irrelevant to the eroded dentin, regardless of the treatment used to control dentin erosive wear, because the condition of the substrate itself makes it less prone to adhesion.^15^

Another explanation would be the pH of the 2% CHX solution. Even if slightly acidic, it may contribute to substrate demineralization during its one-minute application, potentially reducing the effectiveness of resin monomer impregnation.^18,39^ Despite numerous studies evaluating the role of CHX as an antiproteolytic primer, its effect on reducing µTBS to dentin remains controversial.^16^

A systematic review and meta-analysis questioned the applicability of CHX as an antiproteolytic primer in maintaining µTBS to the dentin over long-term aging.^15^ It is well-documented that µTBS to dentin decreases over time because of the degradation of the hybrid layer.^5-7^ Our findings, which evidenced unrestricted µTBS reduction with aging, are in line with the findings of an in vitro study,^40^ and clinical trials have found that CHX use as part of the adhesive treatment did not significantly improve the durability of the restorations.^37,41^

The proteolytic activity in the dentin matrix, related to erosive wear and to the efficacy and longevity of adhesive restorations, underscores the need for continued development of satisfactory and highly conservative therapeutic alternatives. Dentists need to understand the role of these enzymes and consider their inhibition when establishing protocols related to the adhesive restoration of eroded dentin.

The present in vitro study has some limitations, including the inability to accurately replicate intraoral conditions during the erosive challenge and pulpal fluid flow during bonding procedures. Additionally, the study employed only one type of proteinase inhibitor (CHX) in an aqueous solution and only one combination of a simplified etch-and-rinse adhesive plus a nanoparticle resin composite. In light of these limitations, strategies other than noninvasive CHX use for controlling erosive wear or interface aging warrant further investigation, considering that CHX did not influence long-term bonding to eroded dentin.

## Conclusion

CHX application for controlling dentin erosive wear proved not favorable but also not adverse to bond strength to the dentin restored with an etch-and-rinse adhesive combined with a nanoparticle composite. CHX application as part of the dentin adhesive treatment may, however, raise concerns, at least when used as part of the treatment of sound dentin.
